# Characteristics of the Multiplicity of Randomized Clinical Trials for Coronavirus Disease 2019 Launched During the Pandemic

**DOI:** 10.1001/jamanetworkopen.2020.15100

**Published:** 2020-07-13

**Authors:** Ramez Kouzy, Joseph Abi Jaoude, Carolina J. Garcia Garcia, Molly B. El Alam, Cullen M. Taniguchi, Ethan B. Ludmir

**Affiliations:** 1 The University of Texas MD Anderson Cancer Center, Houston

## Abstract

This systematic review examines current randomized clinical trials of therapeutic agents to treat coronavirus disease 2019 (COVID-19).

## Introduction

High-quality evidence generated by appropriately powered and controlled trials is needed to advance care for patients with coronavirus disease 2019 (COVID-19) and those who are susceptible to it.^1,2^ In the midst of the COVID-19 pandemic, multiple similar therapeutic trials are being conducted in parallel, potentially reducing participant accrual across trials. In this systematic review, we characterize the landscape of current COVID-19 trials to better quantify these potential issues.

## Methods

Institutional review board approval of this study was waived because it exclusively used publicly available data without any protected health information. Screening and trial selection adhered to the Preferred Reporting Items for Systematic Reviews and Meta-analyses (PRISMA) reporting guideline.^3^

We performed a data query of the ClinicalTrials.gov registry for interventional trials in any clinical phase regarding COVID-19 on June 8, 2020. Advanced search parameters included *COVID-19*, *SARS-CoV-2*, *2019-nCoV*, *2019 novel coronavirus*, and *severe acute respiratory syndrome coronavirus 2*. Data were analyzed using SPSS statistical software version 26 (IBM Corp). Data analysis was performed in June 2020.

## Results

Our search yielded 674 trials after removing suspended and halted trials (Figure). Most were randomized multigroup studies (562 of 674 trials [83.4%]). Only 479 of 674 randomized trials (71.1%) included a control group deemed to be valid at the time of data curation (including either standard of care or placebo as the control group). Most of the trials assessed treatment of COVID-19 (570 of 674 trials [84.6%]) rather than its prevention (104 of 674 trials [15.4%]). Of randomized studies, only 201 (35.8%) were multicenter trials (Table). Chloroquines were the most commonly tested intervention (132 of 562 randomized trials [23.5%]; 143 trials total). Among the 201 trials accruing in the US alone, the total expected enrollment was 146 688 participants. This included 33 COVID-19 prevention trials with a planned total accrual of 100 746 participants, of which 86 950 participants (86.3%) were planned to accrue to chloroquine-specific COVID-19 prevention trials. Similarly, there were 168 US-accruing COVID-19 treatment trials with a planned total accrual of 45 942 participants, of which 13 542 participants (29.5%) were planned to accrue to chloroquine-specific COVID-19 treatment trials. Primary end points most commonly assessed among randomized studies were time to symptom and sign resolution (212 trials [37.7%]), mortality (180 trials [32.0%]), viral clearance (124 trials [22.1%]), and mechanical ventilation (57 trials [10.1%]) (Table).

**Figure.**
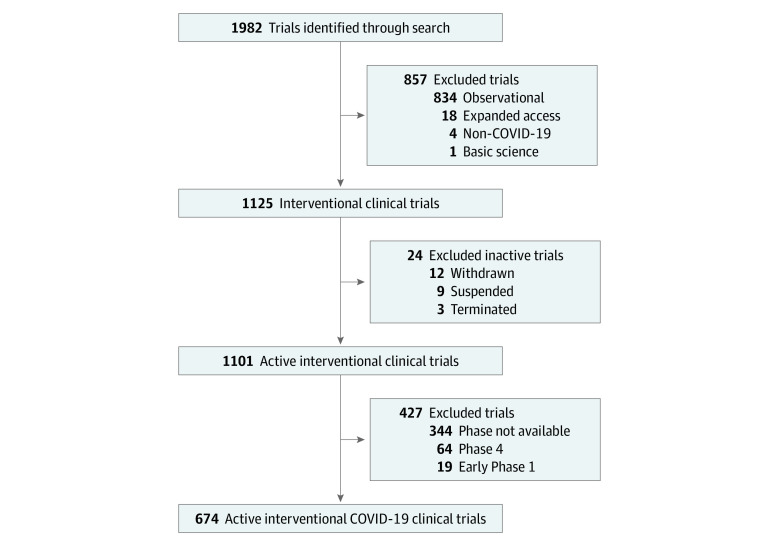
Flowchart of Screening, Eligibility, and Inclusion of Randomized Clinical Trials for Coronavirus Disease 2019 (COVID-19)

**Table.** Characteristics of Coronavirus Disease 2019 Clinical Trials

Characteristic	Trials, No. (%)	
	Randomized (n = 562)	Nonrandomized (n = 112)^a^
Multicenter trials	201 (35.8)	23 (20.5)
Multinational trials	22 (3.9)	1 (0.9)
Any blinding^b^	331 (58.9)	3 (2.7)
Intervention^c^		
Chloroquines^d^	132 (23.5)	11 (9.8)
Biologicals	177 (31.5)	60 (53.6)
Convalescent plasma	30 (5.4)	18 (16.1)
Tocilizumab	21 (3.7)	6 (5.4)
Tyrosine kinase inhibitor	20 (3.6)	12 (10.7)
Antivirals	55 (9.8)	1 (0.9)
Remdesevir	9 (1.6)	0
Protease inhibitors	37 (6.8)	1 (0.9)
Antibiotics	49 (8.7)	5 (4.5)
Azithromycin	40 (7.1)	4 (3.6)
Primary end point^e^		
Time to symptom and sign resolution	212 (37.7)	51 (45.5)
Mortality	180 (32.0)	23 (20.5)
Viral clearance	124 (22.1)	16 (14.3)
Need for mechanical ventilation	57 (10.1)	5 (4.5)
Industry sponsorship	175 (31.1)	19 (17.0)

^a^ Nonrandomized trials included both single-group and nonrandomized multiple-group trials.

^b^ Blinding included single, double, triple, and quadruple blinding.

^c^ Interventions were counted independently as many trials included multiple interventions.

^d^ Chloroquines included hydroxychloroquine and chloroquine.

^e^ Primary end points were counted independently because many trials included multiple primary end points.

## Discussion

We found a high rate of trial multiplicity, particularly with chloroquines, which are being tested in 143 studies. Although these overlapping trials may afford opportunities for replication and validation, the high degree of multiplicity also enhances the likelihood of finding a positive result through chance alone, potentially resulting in widespread use of an ineffective and possibly hazardous intervention.^4^

The fragmentation of efforts could also lead to unnecessary competition for participants, which potentially compromises trial accrual and statistical power for all trials. This worrisome scenario has already occurred in China.^5^ Because the projected participant accrual for US-only COVID-19 treatment trials is approximately 45 942 participants (13 542 to chloroquines alone), it seems unlikely that this target will be achieved given the intrinsic challenges of participant accrual during an active pandemic.

Notably, our study is limited through the use of a single US-based clinical trials registry, potentially representing only a fraction of the worldwide COVID-19–related trials portfolio. Furthermore, the ever-changing landscape of COVID-19 clinical research amid this pandemic may complicate future interpretation of this report.

Although current trials have been initiated with the best intentions, the medical community must be mindful of the potential issues of incomplete participant accrual and publication bias that are introduced by enabling dozens of similar trials simultaneously.^6^ Together, these factors endanger the capacity to rapidly produce meaningful evidence that is vital during this critical time. Avoiding these pitfalls requires coordination of efforts. This could be achieved, in part, through makeshift cooperative groups to improve participant accrual and decrease duplicative efforts. Institutional review boards and regulators (including the US Food and Drug Administration) must also work together to responsibly ease roadblocks to coordinate pooled analyses across trials. These efforts should include synchronization and standardization of end points, focusing on the most meaningful and objective outcomes (eg, all-cause mortality, intensive care unit admission, and mechanical ventilation). It is hoped that these measures will expedite generation of high-quality prospective data to guide effective treatments while maximizing resource allocation.
